# The Effect of Implant Distribution on Functional and Patient‐Reported Outcomes of Mandibular Overdentures Retained by Four Mini Implants

**DOI:** 10.1111/joor.14024

**Published:** 2025-05-09

**Authors:** Jaqueline Ferreira de Sá, Lays Noleto Nascimento, Júlia Fonseca de Moraes Sousa, Thalita Fernandes Fleury Curado, Murali Srinivasan, Gerald McKenna, Martin Schimmel, Cláudio Rodrigues Leles

**Affiliations:** ^1^ School of Dentistry Federal University of Goias Goiania Goias Brazil; ^2^ Clinic of General‐, Special Care‐ and Geriatric Dentistry Center of Dental Medicine, University of Zurich Zurich Switzerland; ^3^ Centre for Public Health Queen's University Belfast Belfast UK; ^4^ Department of Reconstructive Dentistry and Gerodontology School of Dental Medicine, University of Bern Bern Switzerland; ^5^ Division of Gerodontology and Removable Prosthodontics University Clinics of Dental Medicine, University of Geneva Geneva Switzerland

**Keywords:** dental implant, edentulous patient, overdenture

## Abstract

**Purpose:**

This study investigated the effects of implant distribution on functional and patient‐reported outcomes of patients treated with a mandibular overdenture retained by four mini implants.

**Materials and Methods:**

Seventy‐four participants received four titanium–zirconium mini implants in the anterior mandible, and the relative position of the implants was assessed in post‐treatment computed tomography scans with three reference points in the incisal region and first molars of the overdenture. DICOM files were analysed using Blooming Artefact Reduction filters to allow the visualisation of the mini implants in their three‐dimensional position without overlapping bone structures. The implant distribution parameters were the polygon area formed by the four mini implants (implant area), anterior and posterior cantilever extensions, and antero‐posterior spread. Outcomes included anterior and posterior maximum voluntary bite force (MBF), masticatory performance (MP), oral health‐related quality of life impacts (OHIP‐Edent) and satisfaction with the overdenture. Paired *t*‐test, Pearson's correlation test and multiple regression were used for data analysis.

**Results:**

The implant distribution area was the most relevant variable for all functional parameters. The larger the distribution, the better the MP (*p* = 0.003), and the anterior (*p* = 0.011) and posterior MBF (*p* < 0.001). Concerning patient‐reported outcomes, no effect of implant distribution was observed (*p* > 0.05), suggesting that the potential benefit of better distribution may not affect patients' perception of the treatment.

**Conclusion:**

Findings corroborate the influence of implant distribution of the four mini implants on functional parameters and, although the best implant distribution may depend highly on anatomical factors, these parameters should be considered an important prognostic factor for treatment success.

**Trial Registration:**

ClinicalTrials.gov: NCT04760457

## Graphical abstract

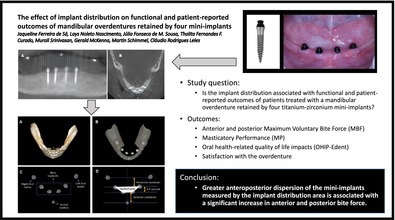

## 1 Introduction

Although the clinical and functional advantages of two‐implant mandibular overdentures over conventional complete dentures are well known, biomechanical problems related to the rotational movements of the overdenture can negatively impact the masticatory capacity of edentulous patients. Kimoto et al. [1] observed that the patients who were aware of rotational movement in their overdentures were significantly less satisfied with their chewing ability than those who felt no rotation. Factors such as the arrangement of the anterior teeth and the extensions of the denture were predictors of the awareness of denture rotation [1]. Moreover, the use of additional implants may reduce the rotation of the overdenture when compared to the use of two implants [2] and therefore, the use of four implants may be a more appropriate design in the mandible, particularly when mini implants are used [3, 4].

However, it is not only the number of implants, but also their position and distribution which may be relevant for the stability of the overdenture under occlusal loading, including rotational movements [5]. Poor implant distribution can overload specific areas of the jaw, may lead to peri‐implant bone loss, and potential long‐term complications [6]. Furthermore, long distal cantilevers can increase the risk of prosthodontic fractures or displacements during mastication and other functional movements. A previous in vitro study showed that the position and distribution of implants in the arch and the mucosal support characteristics may play a relevant role in overdenture stability [7]. However, there is a lack of clinical evidence confirming the effects of the aforementioned factors on the stability of implant‐retained overdentures.

Therefore, this study investigated the effect of the distribution of four mini implants, specifically the anterior–posterior (AP) spread and distribution of the implants, on patient‐reported and functional outcomes for patients treated with a mandibular overdenture opposed by a maxillary conventional complete denture. The study hypothesis was that treatment outcomes are affected by the distribution pattern of mini implants, specifically that better AP spread and larger distribution of the mini implants would result in superior treatment outcome measures.

## 2 Materials and Methods

### 2.1 Study Design and Clinical Intervention

This is an observational study which formed part of a larger randomised clinical trial [8]. The original trial was conducted at the School of Dentistry, Federal University of Goias, Brazil, in accordance with the Declaration of Helsinki on ethical principles for medical research involving human subjects and after approval by the local research ethics committee (CAAE: 79050924.6.0000.5083), and was prospectively registered at ClinicalTrials.gov. The functional outcomes of interest for this study were the maximum voluntary bite force measured in the anterior and posterior regions, and the masticatory performance measured using a mixing‐ability test. Additionally, the influence of implant distribution on patient‐reported outcomes measured as the primary treatment outcome was also assessed.

**FIGURE 1:**
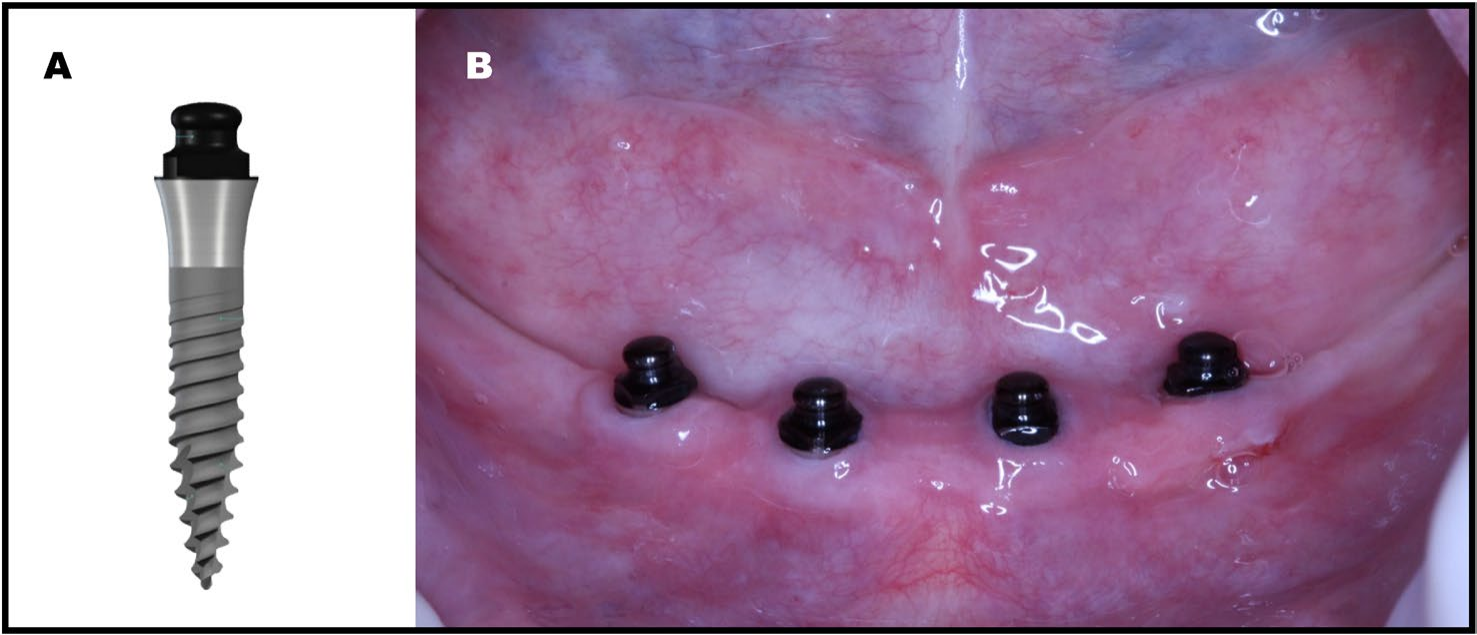
(A) Straumann Mini Implant System. (B) Clinical aspect of the inserted mini implants.

**FIGURE 2:**
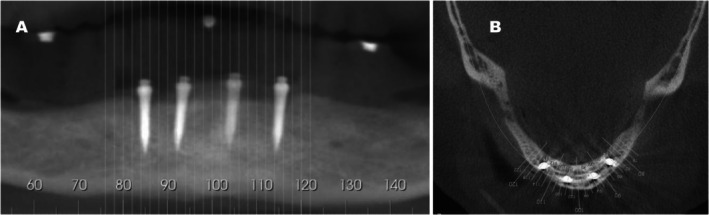
(A) Panoramic CBCT image with the mini implants in position and the three denture teeth landmarks. (B) Sagittal CBCT view.

This retrospective study included patients who had received four one‐piece, titanium–zirconium, apically tapered mini implants with an SLA surface and a carbon‐coated prosthetic connection (Straumann Mini Implant System; Institut Straumann AG, Basel, Switzerland) in the anterior mandible (Figures 1A and 2A) and underwent post‐implant tomography examination. The relative position of the implants was planned to provide an inter‐implant distance of 5 mm, with the most distal implants placed at least 7 mm anterior to the mental foramen bilaterally (Figures 1B and 2B). The four mini implants were placed as parallel as possible and to coincide with the planned path of insertion of the overdenture. The female attachment was composed of a titanium housing with female PEEK matrix inserts (Straumann Optiloc Retentive System; Institut Straumann AG, Switzerland) with two degrees of freedom. The surgical protocol included both flapless and flapped access, and the loading protocol included both immediate and delayed loading, assigned randomly [8]. In addition, the included participants should have complete clinical data available, including functional and patient‐reported outcomes, as well as suitable cone beam computed tomography (CBCT) images that were free from relevant artefacts that impair proper image measurements.

### 2.2 CBCT Acquisition and Analysis

**FIGURE 3:**
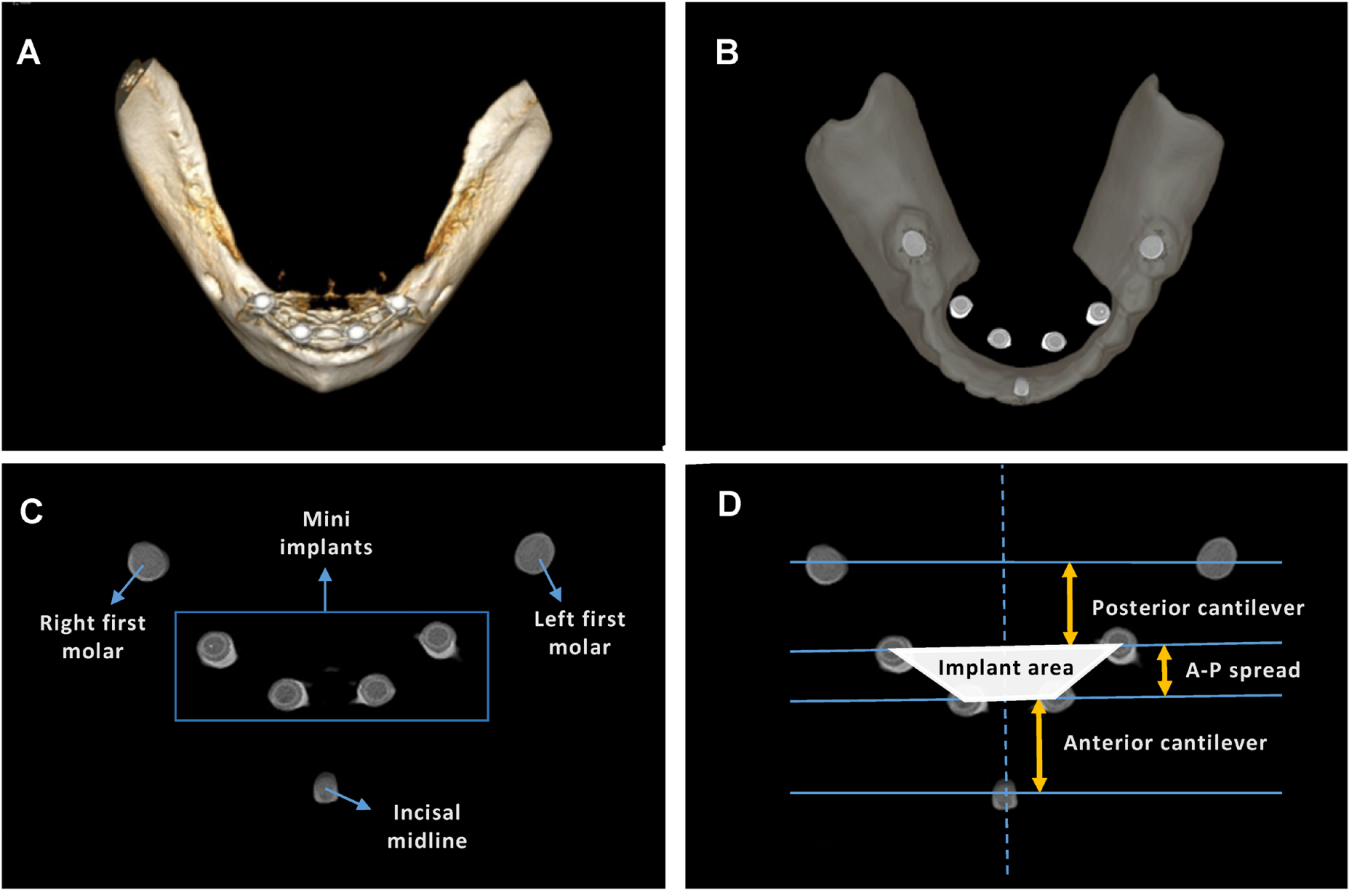
(A) Dimensional reconstruction of the edentulous mandible after treatment with four mini implants. (B) Occlusal view of the superimposition of the denture over the implants after the bone structures filtered out. (C) Occlusal view of the distribution of the mini implants and anterior and posterior teeth landmarks. (D) Measurement of the distribution parameters: Implant area, and anterior and posterior cantilever.

Post‐treatment CBCT scans were obtained for assessment of implant positioning and other image features [Leles et al. 2024]. CBCT exams were acquired using an i‐CAT Precise tomography system (Imaging Sciences International, Hatfield, PA, USA) at a 13‐cm field of view, 0.25‐mm voxel size, 120 kVp, 3.8 mA and 30 s (Figure 3A). The exams were performed with a radiographic guide in position. The guide was fabricated by duplicating the mandibular complete denture and was previously used as a surgical stent to assist with implant positioning. A modification of the radiographic guide was performed by placing three reference points with gutta‐percha, in the incisal region between the lower central incisors, and bilaterally in the central fossae of the first molars, to record the occlusal plane of the prosthesis.

DICOM files were analysed using the e‐Vol DXS software (CDT Software, São José dos Campos, SP, Brazil) on a PC workstation with Windows XP 17 professional SP (Microsoft Corp, Redmond, WA, USA), with an Intel Core 2 Duo 1.86 Ghz‐6300 processor (Intel Corp, Santa Clara, CA, USA) and an EIZO‐Flexscan S2000 monitor, resolution of 1600 × 1200 pixels (EIZONANAO Corp, Hakusan).

The CBCT scans were analysed using the eVol DX software tools and resources; the following filters were applied: (1) BAR 2 filter (Blooming Artefact Reduction) to minimise the imprecision generated in tomography exams with metallic elements and dense materials, which result in white artefacts covering details. The filter also eliminated white artefacts, promoted visualisation of the real size of the analysed area and provided a reduction in magnification to 0% and (2) virtual bone tissue removal filter called “Boneless Implant 2”, which allows visualisation of the mini implants in their three‐dimensional position for analysis without overlapping other bone structures (Figure 3B).

To analyse the implant distribution pattern and the biomechanical geometric variables of the overdenture in relation to the mini implants, a CBCT axial slice was selected at the level of visualisation of the platform centre of the most anterior implant, including the three gutta‐percha points previously marked on the overdenture in the regions of the central incisors and first molars (Figure 3C). Once the section was projected, the “save image in HD” tool was used.

The images were transferred to an image analysis software (Cliniview 10.2.6.5; Instrumentarium), and a measurement equivalent to 10 mm was added using the ruler tool. This image was then transferred to ImageJ software, and the measurement added in Cliniview was selected for pixel per mm calibration. After this step, two calibrated operators (J.F.S. and J.F.M.S.) evaluated all images on an LCD monitor under ideal conditions and with low ambient light, independently and blinded to the patient individual identification. The following parameters were evaluated: area of the polygon formed by the four mini implants (implant area), anterior cantilever extension, AP spread and posterior cantilever extension (Figure 3D).

### 2.3 Dependent Variables and Time Points

The response to overdenture treatment addressed in this study comprised functional and patient‐reported outcomes measured at the 3‐ and 12‐month follow‐up periods. The use of two time points for outcome assessment was utilised in order to model the effect of longitudinal changes in treatment results.

### 2.4 Bite Force and Masticatory Performance

The maximum voluntary bite force (MBF) was measured in Newtons (N) using a digital gnathodynamometer (DMD Kratos) with the prostheses in situ. The device used a bite fork. The thickness of the bite fork is 14.6 mm, and when the bite fork is placed between the teeth and the bite force is applied, the metal rod will deviate and an electric signal is generated and displayed on a digital monitor [9]. MBF was assessed between the upper and lower first molars on both the right and left sides and the incisal region. Participants bit as hard as possible until discomfort was felt, with a 2‐min rest between tests. A contralateral stabiliser was used to prevent displacement of the dentures. The peak force of three consecutive measurements on each side was recorded, and the average was calculated to represent the MBF of each region.

The masticatory performance (MP) was assessed using a bicolor blending test with Hue‐Check Gum (University of Bern, Switzerland). Two pieces of gum, one blue and one pink, were joined together after being moistened. Each patient performed two chewing tests, with 20 cycles respectively, with a 1‐min interval between them to avoid fatigue. After chewing, the gum was washed, dried, sealed and identified. The level of colour blending was analysed by an electronic colorimetric method using ViewGum software (dhal.com, Athens, Greece). The specimens were flattened into 1 mm wafers, scanned at XYZ dpi using a flatbed scanner (HP Photosmart Scanner C4780; Hewlett Packard Corp, Brazil), and the images processed to calculate the circular variance of hue (SD_Hue), where lower values indicate better blending and, therefore, better MP [10].

The detailed methods and methodological aspects of the collection of MBF and MP in this study have been described in detail elsewhere [11].

### 2.5 Dental Patient‐Reported Outcomes

Following overdenture treatment, oral health‐related quality of life (OHRQoL) and patient satisfaction with the mandibular implant‐overdenture (IOD) were assessed using the Brazilian version of the Oral Health Impact Profile for edentulous subjects (OHIP‐Edent) [12] and the McGill Denture Satisfaction Instrument—DSI [13], respectively.

The OHIP‐Edent instrument provided a summary overall score of 19 items rated on a 3‐point Likert‐type scale (0—never; 1—seldom; 2—always). The measured values range from 0 to 38, where higher scores represent worse OHRQoL. The DSI instrument assessed satisfaction covering a set of parameters including general satisfaction, comfort, stability, aesthetics, chewing ability (ease of chewing), ease of cleaning, ability to speak and oral condition, distributed in a 26‐item questionnaire based on a 0–100 visual analog scale (VAS), anchored by the words ‘not at all satisfied’ to ‘extremely satisfied’.

### 2.6 Data Analysis

Data analysis included descriptive statistics, paired *t*‐test, Pearson's correlation test and multiple regression using generalised estimating equations (GEE) for dependent longitudinal data. The reliability of implant distribution measures was assessed by calculating the intraclass correlation coefficient (ICC) between the duplicated measurements.

To run the GEE model for the continuous outcome measures, the analysis was specified as Gamma distribution with log as the link function. Independent factors in the regression model included the implant distribution parameters, anterior and posterior cantilever lengths and study time points. Participants' age and sex were included as control variables. All predictors entered the regression model using the forced entry method to test the main effects of each variable. GEE regression parameter estimates were expressed as the regression coefficients (*B*) and their standard errors. The significance of the model effects was tested using the Wald chi‐squared statistics, and statistical significance was set at *p* < 0.05. For all data analysis, the IBM‐SPSS 24.0 software was used.

## 3 Results

The sample included 74 participants (64.9% female), with a mean age of 64.1 ± 8.0 years, who presented tomographic images free of relevant artefacts, with resolution compatible with the analysis parameters, and complete outcome data.

**FIGURE 4:**
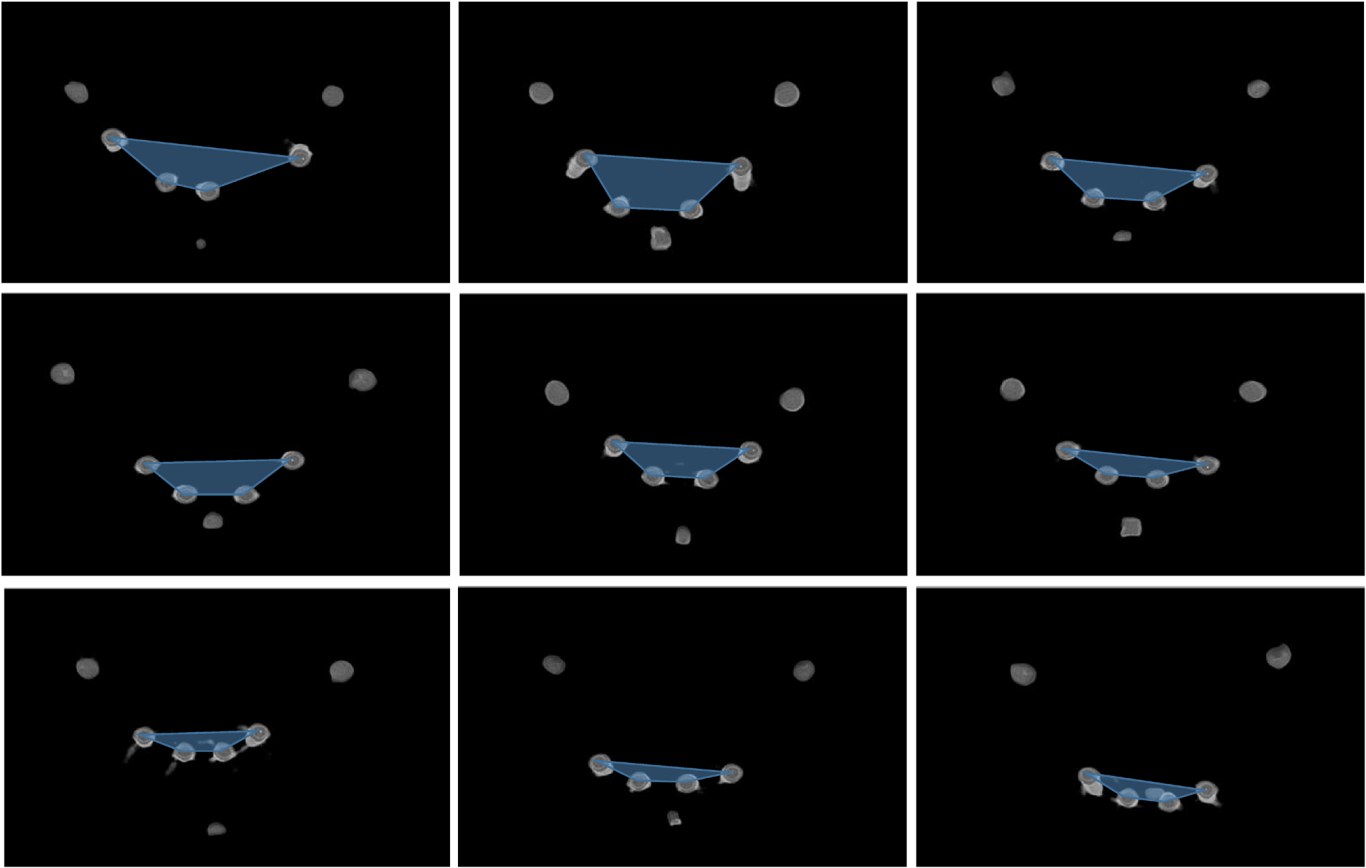
Examples of the implant distribution area of patients included in the study.

Descriptive data in Table 1 details the implant distribution parameters and the functional and patient‐reported outcome measures. A wide variation in the implant area was observed, ranging from 17.3 to 125.3 mm^2^, as shown in Figure 4. The maximum anterior and posterior cantilever values were 10.6 and 15.5 mm, respectively. The ICC values for the implant distribution parameters were all > 0.98, showing excellent consistency between the duplicated measurements.

**TABLE 1:** Summary values of the implant distribution parameters and treatments outcomes measures at the 3‐ and 12‐month follow‐ups.

	Mean (SD)	Minimum	Maximum
Implant distribution parameters			
Implant area (mm^2^)	46.8 (18.0)	17.3	125.3
Anterior cantilever (mm)	4.97 (2.6)	−5.9	10.6
AP spread (mm)	3.48 (0.9)	1.7	6.1
Posterior cantilever (mm)	9.89 (2.3)	4.5	15.5
Outcome measures			
3‐month follow‐up			
Maximum bite force (anterior)	55.9 (27.7)	20.2	177.1
Maximum bite force (posterior)	125.8 (48.0)	36.8	303.9
OHIP‐Edent score	3.01 (4.3)	0.0	25.0
Satisfaction score	92.8 (9.4)	72.8	100.0
12‐month follow‐up			
Maximum bite force (anterior)	67.2 (31.7)	22.4	208.2
Maximum bite force (posterior)	150.2 (62.2)	55.6	443.4
OHIP‐Edent score	1.8 (2.8)	0.0	14.0
Satisfaction score	94.6 (5.3)	72.8	100.0

The measures of treatment outcomes revealed a statistically significant improvement in OHIP‐Edent scores (*p* = 0.003) and patient satisfaction (*p* = 0.070) between the 3‐ and 12‐month follow‐ups. Likewise, MBF increased for both the anterior and posterior bite tests (*p* < 0.001). On the other hand, MP remained stable between the 3‐ and 12‐month follow‐ups (*p* = 0.126).

There was a positive strong correlation between the implant area and the AP spread (*r* = 0.94; *p* < 0.001), and a significant negative correlation between the anterior and posterior cantilever lengths (*r* = −0.78; *p* < 0.001). The length of the anterior cantilever was also inversely correlated with the implant area (*r* = −0.29; *p* = 0.017) and the AP spread (*r* = −0.27; *p* = 0.028). The posterior cantilever length did not correlate with the implant area and the AP spread (*p* > 0.05).

The effects of implant distribution variables on treatment outcomes were tested for each outcome separately. Since no effect of age was observed, and the gender variable did not add value to the interpretation of the models, they were excluded from the final models. Conversely, the time point variable remained, as the time of use of the overdenture tended to improve the measured outcomes at 12 months compared to the initial assessment. Moreover, due to the high correlation between the implant distribution area and the AP spread, the first parameter was included in the model to account for the control of multicollinearity.

Table 2 shows the final regression models representing the association between overdenture treatment outcomes and implant distribution parameters, as affected by the data collection time point. Besides the time point variable, the implant distribution area was the most relevant influential variable for all functional parameters. The larger the distribution area, the better the MP, that is, increased mixing ability (*p* = 0.003), and the anterior (*p* = 0.011) and posterior MBF (*p* < 0.001). Findings also suggest that the length of the anterior and posterior cantilevers had some effect on the MP but no effect on the MBF measured in the anterior or the posterior region.

**TABLE 2:** Regression parameters of the association between overdenture treatment outcomes and implant distribution parameters, as affected by the data collection time point.

Parameter	MP		MBF anterior		MBF posterior		OHIP‐Edent		Satisfaction	
	*B* (SE)	*p*	*B* (SE)	*p*	*B* (SE)	*p*	*B* (SE)	*p*	*B* (SE)	*p*
(Intercept)	0.59 (0.39)	0.134	2.89 (0.69)	< 0.001	4.04 (0.58)	< 0.001	0.098 (3.35)	0.977	4.66 (0.13)	< 0.001
Time point (REF = 3 months)	−0.048 (0.04)	0.167	**0.19** (**0.03**)	**< 0.001**	**0.193** (**0.03**)	**< 0.001**	**−0.477** (**0.166**)	**0.004**	**0.022** (**0.011**)	**0.042**
Implant area	**−0.009** (**0.003**)	**0.003**	**0.009** (**0.003**)	**0.011**	**0.010** (**0.003**)	**0.003**	0.006 (0.016)	0.692	0.001 (0.001)	0.632
Anterior cantilever	**−0.052** (**0.02**)	**0.013**	0.033 (0.04)	0.446	0.028 (0.030)	0.357	0.083 (0.176)	0.639	−0.013 (0.01)	0.135
Posterior cantilever	−0.040 (0.02)	0.051	0.056 (0.04)	0.185	0.016 (0.033)	0.628	0.023 (0.197)	0.909	−0.006 (0.001)	0.464

*Note:* Significant effects are highlighted in bold font.

Concerning the patient‐reported outcomes, no effect of implant distribution parameters was observed (*p* > 0.05), suggesting that the potential benefit of a better implant distribution may not affect the patient's perception of the treatment.

## 4 Discussion

This study investigated the impact of the distribution of four single‐piece mini implants, placed interforaminally, on the biomechanical performance of mandibular IODs. Implant distribution was assessed by means of post‐surgical tomographic analysis, and measurements of maximum bite force and masticatory performance were performed at 3 and 12 months. The central hypothesis was that a better spaced distribution of mini implants would result in better functional performance, which was confirmed by the results. The findings indicate that greater antero‐posterior dispersion of mini implants is associated with a significant increase in anterior and posterior bite force. This measurement is related to an important prognostic indicator [14] and the patient's ability to adequately cut and grind foods of various consistencies.

The use of four mini implants for mandibular IOD retention has been regarded as a suitable clinical treatment option as an alternative to the conventional approach with standard‐diameter implants. The small diameter of the mini implants also allows the insertion without the need for flap elevation or with minimally invasive procedures, often resulting in less surgical burdens and better post‐surgical outcomes [8, 15] and satisfactory 1‐year clinical results.

Alshenaiber et al. [5] demonstrated that the stability of overdentures retained by mini implants is influenced by both the number of mini implants and their strategic distribution. In the study, a greater inter‐implant distance (27 mm) increased resistance to posterior displacement compared to smaller distances (19 mm). Additionally, three widely distributed MDIs, with the two posterior implants positioned in the first premolar region, showed similar resistance to that achieved with four MDIs in the same configuration. This suggests that reducing the number of MDIs can be offset by their strategic distribution. Therefore, while the number of implants is an important factor, careful distribution appears to be even more crucial in enhancing displacement resistance, optimising prosthesis stability and providing greater comfort and functionality to patients.

Especially in the anterior region, the application of forces is often impacted by torque and leverage effects, which can compromise the stability of the overdenture and reduce the maximum force that the patient is able to exert. This result is consistent with previous studies [16], which highlight the relationship between prosthetic stability and incisal bite force, suggesting that improvements in prosthesis retention, through adhesives, can significantly increase patient performance. Furthermore, the posterior bite force may also benefit from a wider implant distribution, although the muco‐implant‐supported nature of the IOD also plays an important role. The alveolar mucosa in the posterior region provides a considerable supporting surface, allowing for better absorption and distribution of applied loads. The combination of strategic implant placement and appropriate mucoimplant support optimises prosthesis stability, reducing discomfort during force application. This results in better MP associated with increased MBF and suggests an overall improvement in oral function parameters [11].

Mini implants, with their one‐piece structure, limit the use of angled components, which prevents the strategy of increasing the polygonal support area with inclined abutments. This is relevant because angled components could improve prosthesis adaptation and retention when implants are not uniformly distributed [17]. Without this possibility, a significantly reduced polygonal support area can result in frequent overdenture displacement under axial and longitudinal forces, compromising functional effectiveness and patient comfort. Therefore, careful biomechanical assessment and precise planning are essential to avoid these problems and ensure the long‐term success of overdentures.

Nevertheless, the results of this study indicate that implant distribution did not significantly influence patient‐reported outcomes, such as satisfaction with the prostheses and quality of life. This suggests that although optimised implant distribution may improve biomechanical aspects, such as stability and retention, these benefits do not necessarily translate into the patient's subjective perception of the treatment. A study comparing 2, 3 and 4 implants with different attachment types concluded that prostheses supported by four implants resulted in higher quality of life scores, while patient satisfaction was not influenced by the number of implants or the type of attachment used [18].

However, the lack of significant differences in outcomes reported in this study can be explained by several factors. Quality of life and patient satisfaction are complex phenomena shaped by a combination of aspects beyond prosthetic functionality, such as personal expectations, psychological comfort and social support [19]. Additionally, treating edentulous individuals with implant‐retained overdentures can effectively reduce complaints associated with conventional dentures. This alone can address the primary needs of patients, regardless of variations in implant distribution. A long‐term study indicated that overall satisfaction with implant‐retained mandibular overdentures remained high after 8 years of treatment, regardless of the treatment strategy employed, reinforcing our findings [20]. Another point to consider is that the participants in this study were treated in a university setting where they did not pay for their treatment; this environment may have also contributed to a lower dissatisfaction rate.

Moreover, a ceiling effect was observed in the satisfaction and OHRQoL data, in which a large percentage of participants achieved the best or maximum possible response. This was particularly problematic for the purpose of this study since it made it difficult to differentiate between participants at the top of the scale due to the slight variance in the data and the restricted range of values that the outcome variable could take. Therefore, a highly positive perception of treatment benefits may occur regardless of different implant distribution patterns. Finally, in addition to standard instruments with sufficient psychometric properties and high methodological quality and comparability, such as the Oral Health Impact Profile (OHIP), the use of further assessment of specific treatment outcomes, individual questions, or tailored questionnaires can be incorporated into future studies to ensure specificity to the instrument that addresses the patient's perception of the IOD function and use [21].

Another significant limitation of this study is the absence of a detailed and validated clinical test on the risk of denture displacement during mastication. Although we observed a positive correlation between greater antero‐posterior dispersion of mini implants and increased MBF and MP, the real impact of this distribution on denture stability in everyday use situations remains unclear. Further investigations with clinical tests of denture displacement during mastication are needed to determine how these variables interact in practice and to provide a more solid basis for clinical decision‐making and more accurate prognoses.

## 5 Conclusion

Within the limits of this observational study, the findings reported corroborate the influence of implant distribution in improving functional parameters in patients treated with a mandibular overdenture retained by four mini implants. Nevertheless, although the best implant distribution may depend highly on anatomical factors, this parameter should be considered an important prognostic factor for treatment success.

## Author Contributions

**Jaqueline Ferreira de Sá:** data collection and investigation, data analysis and interpretation, original draft preparation, project administration. **Lays Noleto Nascimento:** data collection and investigation, data analysis and interpretation, original draft preparation. **Júlia Fonseca de Moraes Sousa:** data collection and investigation, data analysis and interpretation, original draft preparation. **Thalita Fernandes Fleury Curado:** data collection and investigation, data analysis and interpretation, original draft preparation. **Murali Srinivasan:** conceptualisation, methodology, investigation, draft writing – review and editing. **Gerald McKenna:** data analysis and interpretation, original draft writing – review and editing, funding acquisition. **Martin Schimmel:** data analysis and interpretation, original draft writing – review and editing, funding acquisition. **Cláudio Rodrigues Leles:** conceptualisation, methodology, statistics, original draft writing – review and editing, funding acquisition, project administration.

## Conflicts of Interest

Cláudio Rodrigues Leles received a grant from the International Team for Implantology (ITI) to conduct this study (Grant 1447_2019) and received the mini implants, components and instruments from Institut Straumann AG, Switzerland. Cláudio Rodrigues Leles, Murali Srinivasan, Martin Schimmel and Gerald McKenna are the recipients of other funding from Institut Straumann AG and the ITI. The other authors do not report any conflicts of interest related to the present study.

## Peer Review

The peer review history for this article is available at https://www.webofscience.com/api/gateway/wos/peer‐review/10.1111/joor.14024.

## Data Availability

The data that support the findings of this study are available from the corresponding author upon reasonable request.
